# Interstitial Ag^+^ Engineering Enables Superior Resistive Switching in Quasi-2D Halide Perovskites

**DOI:** 10.3390/nano15161267

**Published:** 2025-08-16

**Authors:** Haiyang Qin, Zijia Wang, Qinrao Li, Jianxin Lin, Dongzhu Lu, Yicong Huang, Wenke Gao, Huachuan Wang, Chenghao Bi

**Affiliations:** 1 College of Intelligent Systems Science and Engineering, Harbin Engineering University, Harbin 150001, China; 23qhy@hrbeu.edu.cn (H.Q.); linjianxin@hrbeu.edu.cn (J.L.);; 2 Qingdao Innovation and Development Center of Harbin Engineering University, Harbin Engineering University, Qingdao 266500, China; 3 Sanya Nanhai Innovation and Development Base of Harbin Engineering University, Harbin Engineering University, Sanya 572024, China; 4 State Key Laboratory of Advanced Marine Materials, Key Laboratory of Marine Environmental Corrosion and Bio-Fouling, Institute of Oceanology, Chinese Academy of Sciences, Qingdao 266000, China; 5 Yantai Research Institute, Harbin Engineering University, Yantai 264000, China

**Keywords:** quasi-2D halide perovskites, interstitial engineering, memristor, neuromorphic devices

## Abstract

Halide perovskite-based memristors are promising neuromorphic devices due to their unique ion migration and interface tunability, yet their conduction mechanisms remain unclear, causing stability and performance issues. Here, we engineer interstitial Ag^+^ ions within a quasi-two-dimensional (quasi-2D) halide perovskite ((C_6_H_5_C_2_H_4_NH_3_)_2_Cs_*n*−1_Pb_*n*_I_3*n*+1_) to enhance device stability and controllability. The introduced Ag^+^ ions occupy organic interlayers, forming thermodynamically stable structures and introducing deep-level energy states without structural distortion, which do not act as non-radiative recombination centers, but instead serve as efficient charge trapping centers that stabilize intermediate resistance states and facilitate controlled filament evolution during resistive switching. This modification also leads to enhanced electron transparency near the Fermi level, contributing to improved charge transport dynamics and device performance. Under external electric fields, these Ag^+^ ions act as mobile ionic species, facilitating controlled filament formation and stable resistive switching. The resulting devices demonstrate exceptional performance, featuring an ultrahigh on/off ratio (∼10^8^) and low operating voltages (∼0.31 V), surpassing existing benchmarks. Our findings highlight the dual role of Ag^+^ ions in structural stabilization and conduction modulation, providing a robust approach for high-performance perovskite memristor engineering.

## 1. Introduction

In recent years, neuromorphic computing has emerged as a transformative approach to overcome the fundamental limitations of von Neumann architectures, prompting an urgent demand for next-generation electronic components capable of emulating biological synapses [1]. Among these, memristors based on resistive switching mechanisms have attracted intense interest as non-volatile memory elements [2], offering advantages such as structural simplicity, nanoscale footprint, ultralow power consumption, and seamless CMOS compatibility [3,4,5]. Notably, halide perovskite-based memristors exhibit unique ionic migration dynamics [6], highly tunable electronic structures [7], and excellent solution-processability [8]—traits that collectively position them as compelling candidates for scalable, energy-efficient neuromorphic hardware.

Memristor technologies have diversified into various types, notably including interface-engineered devices [9] and filamentary memristors that rely on localized conductive path formation [10,11]. Each architecture exhibits distinct switching mechanisms and characteristics. Among them, filamentary-type memristors are particularly attractive due to their high switching ratios, excellent scalability, and ability to emulate synaptic plasticity. Quasi-two-dimensional (quasi-2D) halide perovskites, with their alternating organic–inorganic layered structures, offer structural flexibility and the potential for simultaneous modulation of band structure and defect states via molecular design. Compared to their three-dimensional counterparts, their reduced dimensionality provides stronger spatial confinement, which effectively regulates ion migration. These features have driven their increasing prominence in both optoelectronic and memristive research [12,13,14,15]. However, despite their favorable layered architectures, quasi-2D halide perovskites still face challenges such as unstable ion migration, limited defect tolerance, and incomplete understanding of their switching dynamics—all of which constrain performance and hinder practical adoption. Recent efforts have explored the incorporation of metal cations to address these issues. For instance, Chen et al. employed Cu^2+^ with Tween (polyethylene glycol sorbitan monooleate) to assist crystallization in light-emitting applications, yet failed to obtain well-formed copper perovskite thin films [16]. Gao et al. modulated Li^+^ concentrations to reduce surface roughness, although the underlying switching mechanism remained unverified [17]. Pérez-Martínez introduced oxidized AgI as an interfacial layer to inject Ag^+^ during initial switching, resulting in reduced set voltages, though the enhancement in switching ratio remains mechanistically ambiguous [18]. These unresolved issues pose major obstacles to the widespread application of halide perovskite memristors.

Herein, we report a novel interstitial-site engineering strategy for quasi-2D halide perovskite memristors based onPEA_2_Cs_*n*−1_Pb_*n*_I_3*n*+1_, where Ag^+^ ions are introduced not via conventional substitutional doping or interfacial diffusion, but through intercalation into the van der Waals gaps of the layered structure. This approach leverages the unique structural confinement of quasi-2D perovskites to spatially localize Ag^+^ ions within organic interlayers, forming energetically favorable interstitial reservoirs that actively participate in conductive filament formation under an electric field. In contrast to previously reported interface-modified or electrode-injected Ag-based systems, our design enables intrinsic ion regulation through structural engineering, thereby enhancing switching uniformity and device controllability. First-principles density functional theory (DFT) calculations confirm the thermodynamic preference for interlayer Ag^+^ incorporation, while experimental characterizations reveal improvements in interfacial carrier transport, reduced trap density, and more stable resistive switching behavior. As a result, the fabricated devices demonstrate superior performance metrics, including an ultralow set voltage (∼0.31 V) and an ON/OFF current ratio as high as 10^8^, outperforming most state-of-the-art quasi-2D perovskite-based memory devices. This work establishes a new materials design paradigm that exploits interstitial ion engineering to unlock the full potential of layered perovskites for high-performance, energy-efficient memristive and neuromorphic applications.

## 2. Results and Discussion

### 2.1. Structural and Morphological Characterization

A one-step solution-processing method was adopted, wherein PEAI, CsI, and PbI_2_ were co-dissolved in DMF and stirred for 12 to 24 h. The resulting precursor solution was spin-coated onto pre-patterned electrodes under an inert atmosphere to fabricate quasi-two-dimensional (quasi-2D) perovskite films (Control). To enhance ion migration-mediated resistive switching behavior, Ag^+^ ions were introduced as a functional additive into the precursor solution, producing Ag^+^-incorporated films under otherwise identical fabrication conditions.

**Figure 1:**
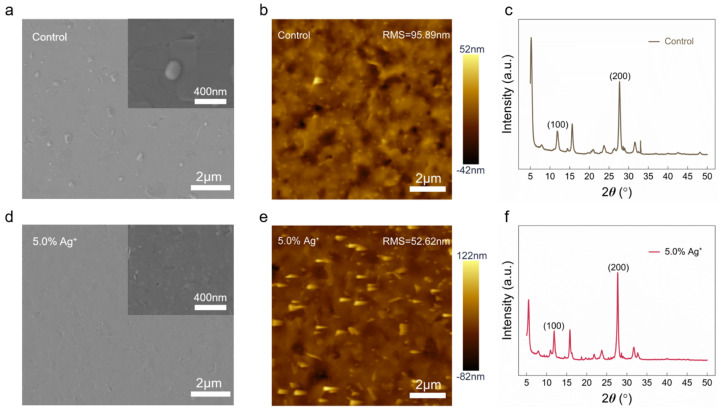
(**a**,**d**) Top-view SEM images of control (**a**) and 5.0% Ag^+^-incorporated (**d**) perovskite films. (**b**,**e**) AFM surface topographies of control (**b**) and 5.0% Ag^+^-incorporated (**e**) films. (**c**,**f**) XRD patterns of control (**c**) and 5.0% Ag^+^-incorporated (**f**) films.

The surface morphologies of the control and Ag^+^-modified PEA_2_Cs_*n*−1_Pb_*n*_I_3*n*+1_ perovskite films were examined using scanning electron microscopy (SEM), as shown in Figure 1a,d. Compared to Ag^+^-free films, those containing Ag^+^ exhibited significantly reduced pinhole density and improved film continuity. With increasing Ag^+^ concentration, SEM images (Figure S1) revealed progressive Ag^+^ incorporation into the lattice, where it coordinated with PbI_6_ and contributed to a more compact and uniform morphology. At lower concentrations, Ag^+^ preferentially occupied the organic interlayers of the quasi-2D perovskite lattice. However, excessive Ag^+^ led to film degradation, including increased surface roughness, void formation, and inhomogeneous grain distribution.

To quantitatively evaluate the densification effect of Ag^+^ on PEA_2_Cs_*n*−1_Pb_*n*_I_3*n*+1_ perovskite films, atomic force microscopy (AFM) was performed to measure surface roughness. Unless otherwise noted, “5.0% Ag” refers to a stoichiometric ratio of Pb:Ag = 20:1. The film incorporating Ag^+^ exhibited a reduced root-mean-square (RMS) roughness of 52.62 nm (Figure 1e), compared to 95.89 nm for the control sample (Figure 1b), indicating improved surface smoothness upon Ag^+^ incorporation. To further optimize the Ag^+^ content, films with varying concentrations were examined by AFM, as shown in Figure S2. At a low incorporation level of 1.0% Ag^+^ (Pb:Ag = 100:1), the RMS roughness markedly increased to 110.3 nm, suggesting hindered grain coalescence and insufficient film densification. Conversely, increasing the Ag^+^ content to 10.0% (Pb:Ag = 10:1) led to a moderate RMS increase to 68.67 nm, indicative of morphological degradation likely arising from agglomeration and void formation. These results collectively reveal a non-monotonic trend, wherein moderate Ag^+^ incorporation optimizes the crystallization process and yields the smoothest and most compact film morphology, while both under- and over-incorporation lead to increased roughness and structural disorder. Additionally, energy-dispersive X-ray spectroscopy (EDS) was employed to compare elemental distributions before and after Ag^+^ introduction. The corresponding elemental maps (Figures S3 and S4) confirm enhanced film uniformity and compositional homogeneity following Ag^+^ incorporation.

To assess the effect of Ag^+^ incorporation on crystal structure, X-ray diffraction (XRD) measurements were conducted on PEA_2_Cs_*n*−1_Pb_*n*_I_3*n*+1_ films before and after Ag^+^ incorporation. As shown in Figure 1c,f, the primary diffraction peak positions remain unchanged, confirming that Ag^+^ incorporation does not alter the perovskite phase. However, the intensity of the (100) and (200) reflections increases markedly after Ag^+^ treatment, with noticeably sharper peak shapes, indicating enhanced crystallographic orientation and improved structural ordering [19]. Additionally, the full width at half maximum (FWHM) of the dominant diffraction peaks narrows from 0.44 to 0.33, suggesting Ag^+^ promotes grain growth and reduces internal strain. These improvements contribute to overall enhanced crystallinity, which facilitates more efficient charge transport and improved interfacial stability. Additionally, two weak diffraction peaks appear after Ag^+^ incorporation, indicative of low-n quasi-2D phase formation (e.g., PEA_2_PbI_4_ or PEA_2_Cs_1_Pb_2_I_7_), which generally exhibit larger interplanar spacing and enhanced stacking order. This phase evolution further supports the enhancement of lattice periodicity and long-range structural coherence, consistent with prior observations in layered perovskite systems [20].

### 2.2. Charge Carrier Dynamics

**Figure 2:**
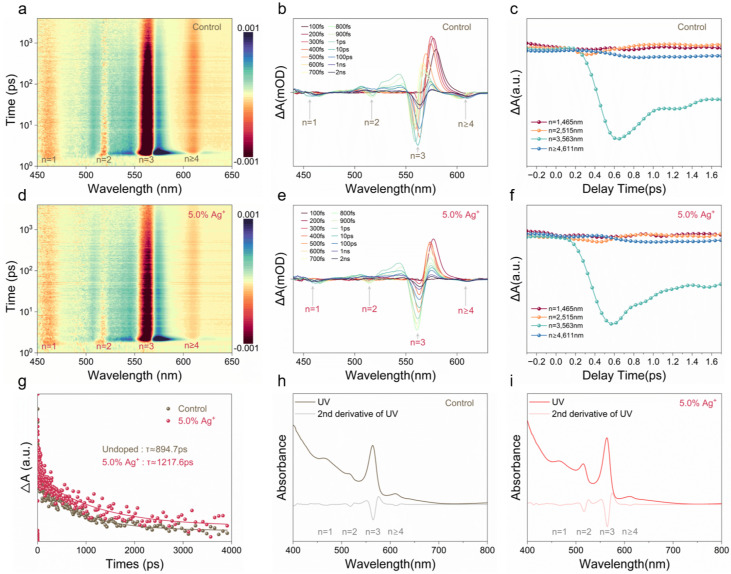
(**a**–**c**) Transient absorption (TA) maps, spectral evolution, and kinetic decay curves at 563 nm for the control perovskite film. (**d**–**f**) Corresponding TA characterizations for the 5.0% Ag^+^-incorporated film. (**g**) Bi-exponential fitting of kinetic decay curves at 563 nm for both films. (**h**,**i**) UV–vis absorption spectra and their second derivatives for the (**h**) control and (**i**) 5.0% Ag^+^-incorporated films.

Transient absorption spectroscopy (TAS) was employed to investigate the photo-induced carrier dynamics and recombination behavior of quasi-two-dimensional (quasi-2D) perovskite thin films. Two-dimensional pseudocolor TA maps acquired under different excitation wavelengths are shown in Figure 2a,d. Distinct ground state bleach (GSB) bands at 450–470, 505–520, 550–580, and >600 nm correspond to carrier populations in phases with layer numbers n=1, 2, 3, 4, and *∞*, respectively [21,22]. To probe carrier dynamics, spectral evolution was monitored in the 450–650 nm range with delay times spanning 100 fs to 2 ns. As shown in Figure 2b,e, energy transfer pathways between low-*n* and high-*n* phases were resolved. Control films exhibit characteristic GSB peaks at 465 nm (n=1), 515 nm (n=2), 563 nm (n=3), and 611 nm (n≥4). Following Ag^+^ incorporation, the peak positions remain unchanged, but signals corresponding to n=2 and 3 are markedly enhanced. This pronounced intensity enhancement indicates that Ag^+^ modulates the crystallization kinetics and facilitates the preferential formation of low-n phases with better vertical alignment. Moreover, the spectral redistribution implies a more complete energy transfer from the *n* = 1 phase toward the more stable *n* = 2 and 3 phases, contributing to improved charge transport in quasi-2D perovskite-based memristive devices.

In the control samples, GSB signals for n=2 and 3 decay significantly over time, whereas those for n≥4 remain stable, indicating preferential carrier accumulation in higher-*n* domains. In contrast, Ag^+^-incorporated films (Figure 2c,f) exhibit stronger and more sustained GSB signals, particularly at 563 nm (n=3), where ΔA decays more rapidly (0.57 ps vs. 0.66 ps in control), suggesting faster charge funneling. These results indicate that Ag^+^ facilitates directional energy transfer from low-*n* to high-*n* phases, potentially enhancing charge extraction and overall carrier transport efficiency [23].

With increasing delay time, the GSB signals for n=2 and 3 decay progressively, while those for n≥4 remain largely stable, corroborating the existence of efficient energy transfer from high-energy (low-*n*) to low-energy (high-*n*) phases. In comparison to the control, the GSB_*n*=2_ and GSB_*n*=3_ features in 5.0% Ag^+^ films exhibit more rapid decay, indicating shortened carrier lifetimes and enhanced energy transfer. Notably, the average carrier lifetime is extended to 1217.6 ps in 5.0% Ag^+^modified films, compared to 894.7 ps in control samples, likely attributable to defect passivation by Ag^+^ that suppresses non-radiative recombination pathways [24]. The kinetic traces shown in Figure 2g were extracted at the GSB_*n*=3_ position, where the signal quality and fitting convergence were found to be most reliable. The corresponding bi-exponential fitting parameters and average lifetimes are summarized in Table S1. Although other GSB features (n=1,2,≥4) are also visible, they were not used for lifetime extraction due to weaker signals and unsatisfactory fitting performance. Figure 2h,i show UV–vis absorption spectra before and after Ag^+^ incorporation, where a reduction in defect-related absorption is observed. The presence of well-defined absorption peaks corresponding to n=1, 2, 3, and ≥4 indicates the coexistence of multiple quasi-2D phases. The relative enhancement of *n* = 2 peaks after Ag^+^ incorporation suggests a redistribution toward energetically favorable low-dimensional phases rather than a uniform increase in phase purity. This suggests a higher fraction of low-dimensional perovskite phases and reduced phase intermixing in the Ag-incorporated films. Lower-*n* phases (n=1–3) are associated with strong quantum confinement and excitonic effects, which enhance carrier localization and extraction [25]. In contrast, higher-*n* phases (n≥4) facilitate rapid charge transport and extraction, underpinning the fast switching behavior observed in 5.0% Ag^+^ devices. These findings may explain why the memristors presented here predominantly exhibit filamentary-type rather than interface-controlled switching.

### 2.3. Density Functional Theory Analysis

**Figure 3:**
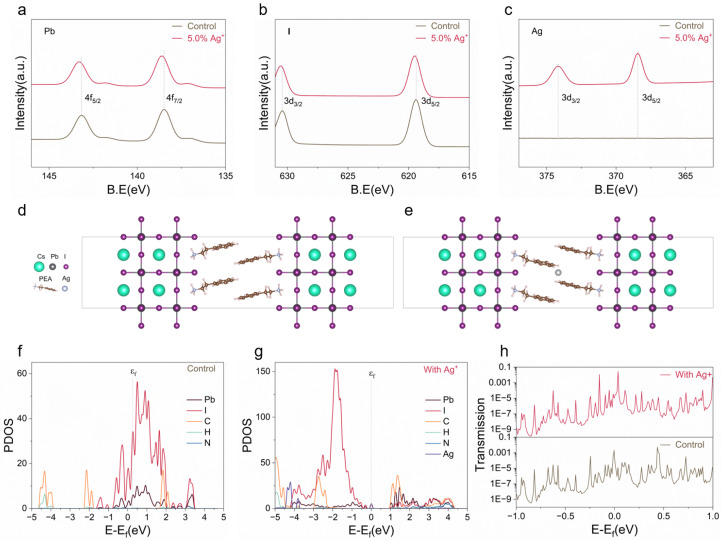
(**a**–**c**) High-resolution XPS spectra of Pb 4f (**a**), I 3d (**b**), and Ag 3d (**c**) for control and 5.0% Ag^+^-incorporated perovskite thin films. (**d**,**e**) Atomic element legend and side-view schematic diagrams of the PEA_2_Cs_*n*−1_Pb_*n*_I_3*n*+1_ lattice (**d**) without and (**e**) with Ag^+^ incorporation. (**f**,**g**) Projected density of states (PDOS) of the quasi-2D perovskite without (**f**) and with (**g**) interstitial Ag^+^ incorporation. (**h**) Transmission spectra of control and Ag^+^-incorporated perovskite systems.

Figure 3a–c presents high-resolution X-ray photoelectron spectroscopy (XPS) spectra for Pb, I, and Ag in perovskite films before and after Ag^+^ incorporation. In the absence of Ag^+^, Pb 4f_5/2_ and 4f_7/2_ peaks are located at 143.11 and 138.48 eV, respectively. Upon Ag^+^ incorporation, these peaks shift to 143.28 and 138.62 eV, indicating an increase in binding energy. A similar trend is observed for I 3d_3/2_ and I 3d_5/2_, which shift from 630.39 and 619.40 eV in the control (the schematic illustration of its structure is shown in Figure 3d) samples to 630.54 and 619.49 eV after Ag^+^ incorporation [26]. As shown in Figure 3c, two distinct peaks corresponding to Ag 3d_3/2_ and Ag 3d_5/2_ are observed at 374.02 eV and 368.02 eV, respectively, in the Ag^+^-introduced samples, whereas no corresponding signals appear in the control group. These newly emerged Ag-related signals further confirm the successful introduction of Ag^+^ species into the perovskite films. These binding energy shifts suggest a stronger interaction environment for both Pb and I atoms in Ag^+^-incorporated films. These positive shifts in binding energy indicate a strengthened local coordination environment between Pb and I atoms, despite Ag^+^ not entering the inorganic lattice directly. The interstitial Ag^+^ ions, primarily located within the organic PEA spacer layers, modulate the electronic structure at the organic–inorganic interface. This interaction indirectly enhances the Pb–I bonding via electronic polarization and electrostatic interaction, leading to reduced lattice distortion and suppressed defect formation. Such interfacial regulation plays a vital role in improving the structural stability of quasi-2D perovskites and contributes to superior memristive performance.

To further elucidate the influence of Ag^+^ spatial distribution on the electronic structure and migration characteristics of quasi-2D perovskites, density functional theory (DFT) calculations were performed, modeling Ag^+^ at lattice-interior (Figure S5) and interlayer interstitial (Figure 3e) positions. Energy comparisons indicate that surface (interfacial) Ag^+^ is thermodynamically favored, with a system energy of $1.048\times 10^{3}$ eV compared to $1.207\times 10^{3}$ eV for the bulk interstitial configuration, corresponding to a substantial energy barrier of 159 eV. This thermodynamic preference implies that Ag^+^ ions predominantly occupy interfacial regions, where local electric fields facilitate their migration and promote filament initiation at lower operating voltages. Moreover, the spatial confinement of Ag^+^ migration pathways enhances the controllability of conductive filament formation, reduces set voltages, and contributes to improved device performance.

Further projected density of states (PDOSs) calculations (Figure 3f,g) reveal that Ag^+^ incorporation predominantly introduces deep states in the range of −3 to −5 eV, without generating significant shallow traps near the band edges. When Ag^+^ resides in the organic interlayer, the corresponding stable states remain electronically inactive under equilibrium, but can migrate under applied bias to serve as sources for metallic filament formation [27]. These deep-level states act as effective charge-trapping centers, enabling the stabilization of intermediate resistance states and facilitating controlled filament growth and rupture. Notably, these deep states do not introduce non-radiative recombination pathways nor interfere with carrier transport, thereby avoiding carrier quenching and contributing to enhanced electronic performance. These findings, together with the observed ultrahigh on/off ratios (∼10^8^) at low operating voltages of ∼0.31 V, further substantiate the cooperative enhancement in device performance attributed to Ag^+^ migration.

Electronic transport simulations provide additional validation. As shown in Figure 3h, devices with interfacial Ag^+^ exhibit significantly higher electron transparency, with peak transmission coefficients approaching $10^{-3}$—nearly two orders of magnitude greater than those of the control (∼10^−5^). This enhancement is attributed to the Ag^+^-induced suppression of defect-mediated scattering and resonance states at organic–inorganic interfaces, thereby promoting more uniform conduction pathways and improved charge transport. Collectively, the PDOS and transport analyses indicate that interstitial Ag^+^ not only optimizes electronic properties in quasi-2D perovskite memristors but also plays a pivotal role in modulating SET/RESET switching dynamics.

### 2.4. Memristor Device Performance Optimization

**Figure 4:**
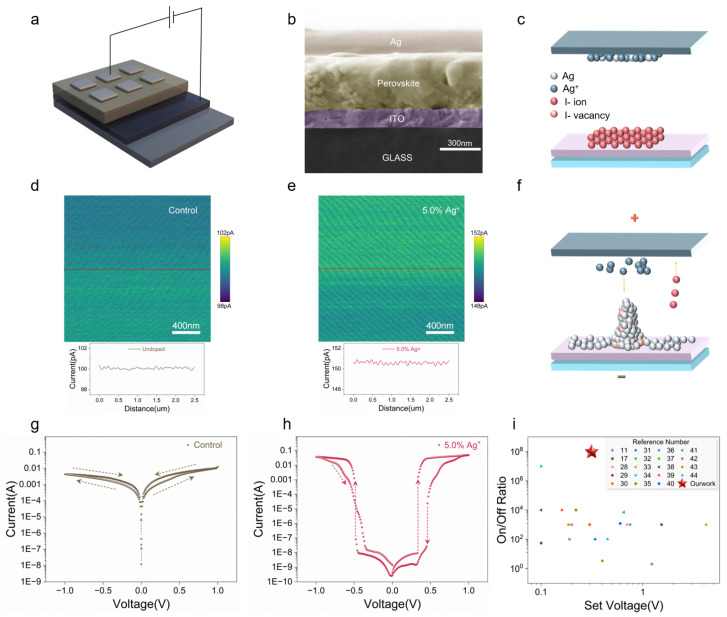
(**a**) Schematic illustration of the Ag/PEA_2_Cs_*n*−1_Pb_*n*_I_3*n*+1_:Ag/Glass memristor device structure. (**b**) Cross-sectional SEM image of the device. (**c**) Schematic of the initial (reset) state in the resistive switching process. (**d**,**e**) C-AFM current maps of control and 5.0% Ag^+^-incorporated perovskite films, respectively. (**f**) Schematic illustration of conductive filament formation during switching. (**g**,**h**) Current–voltage (I–V) characteristics of control and 5.0% Ag^+^-incorporated devices, respectively. (**i**) Benchmark comparison of ON/OFF ratio and set voltage with other halide perovskite memristors.

Figure 4a depicts the structure of the fabricated memristor device, which comprises an Ag/PEA_2_Cs_*n*−1_Pb_*n*_I_3*n*+1_:Ag/ITO/Glass stack. The cross-sectional morphology, shown in Figure 4b, reveals that the perovskite layer is approximately 350 nm thick, with Ag top electrodes (TE) and ITO bottom electrodes (BE) measuring roughly 100 nm and 150 nm, respectively. In the optimized Ag/PEA_2_Cs_*n*−1_Pb_*n*_I_3*n*+1_:Ag/ITO/Glass devices, the memristive switching mechanism—Ag filament formation and rupture—is schematically illustrated in Figure 4c,f [28]. Figure 4c presents the initial (reset) structural state of the device under zero bias, where a certain number of Ag^+^ ions and Ag vacancies are distributed near the ITO/perovskite and Ag/perovskite interfaces. Upon application of a positive bias to the Ag TE (and negative to the ITO BE), Ag atoms at the top electrode are oxidized, releasing electrons and forming Ag^+^ ions. These ions migrate through the perovskite layer toward the ITO BE, where they are reduced to metallic Ag, initiating filament growth from the bottom electrode (as shown in Figure 4d). Continued positive bias drives further upward filament extension, forming multiple conductive pathways and switching the device from a high-resistance state (HRS) to a low-resistance state (LRS), as depicted in Figure 4f. Reversing the bias polarity causes the Ag filaments to undergo oxidation and dissolution at the Ag TE, resulting in the rupture of the conductive pathways and restoration of the HRS. This process completes a full SET/RESET cycle, returning the device to the initial state illustrated in Figure 4c.

For further insight into the switching mechanism, Ag/PEA_2_Cs_*n*−1_Pb_*n*_I_3*n*+1_: Ag/Glass half-devices were fabricated on glass substrates. The schematic device design is presented in Figure S6, and an optical image of the actual device is shown in Figure S7. The Ag electrode dimensions are 4 mm × 1.5 mm, with a central region width of approximately 150 μm. During operation, a forward bias of 32 V was applied to the sample. After 60 min, phase-contrast microscopy revealed the formation site of a conductive filament. Upon further examination at 120 min, multiple additional filament-like features were observed (Figure S8). These observations further substantiate the pivotal role of Ag in the formation of conductive filaments.

To elucidate the impact of Ag^+^ on the electrical properties of perovskite films, conductive atomic force microscopy (C-AFM) measurements were performed at a +0.1 V bias. Figure 4d and Figure 4e present current maps and corresponding line profiles for films with and without Ag^+^, respectively. Under identical conditions, Ag^+^-incorporated films exhibited an average current of 150 pA, a 50% increase relative to the control samples. In addition, cyclic voltage sweeps (0 → +1 V → 0 V → –1 V → 0 V) were used to assess device on/off ratios. As shown in Figure 4g (control) and Figure 4h (5.0% Ag^+^), control devices displayed weak resistive switching (on/off ratio < 10), while devices with 5.0% Ag^+^ achieved a maximum on/off ratio of $10^{8}$. This performance approaches the upper threshold reported for quasi-2D perovskite-based memristors. Remarkably, Ag^+^ incorporation consistently yielded on/off ratios approaching $10^{8}$, underscoring the substantial enhancement in device performance.

Mechanistic analysis further revealed that Ag^+^ incorporation not only passivates defects but also increases the density and mobility of mobile Ag ions, enabling more controlled filament formation and dissolution. Devices with Ag^+^ exhibited set voltages as low as 0.31 V; lower set voltages correspond to reduced switching energy, which is highly advantageous for neuromorphic and energy-efficient memory applications. Furthermore, low operational voltages minimize Joule heating losses, contributing to improved device endurance and stability. To contextualize these results, Figure 4i benchmarks our device’s on/off ratio and set voltage against other metal-incorporated perovskite [11,17,28,29,30,31,32,33,34,35,36,37,38,39,40,41,42,43,44], with additional comparative data summarized in Table S2. Notably, our device occupies the upper-left region of the performance map, achieving an ultrahigh on/off ratio of ∼10^8^ at a remarkably low set voltage of ∼0.31 V. This performance clearly exceeds that of most reported counterparts, which generally suffer from either elevated switching voltages or limited switching contrast. The simultaneous realization of low-voltage operation and high resistance switching ratio underscores the efficacy of interstitial Ag^+^ modulation and positions our device among the most advanced quasi-2D perovskite memristors reported to date.

## 3. Conclusions

This study systematically investigates the modulation of memristive performance in quasi-2D perovskite devices through Ag^+^ incorporation and proposes a dual-pathway enhancement strategy combining structural optimization and device-level conduction tuning. Integrating experimental findings with first-principles calculations, we demonstrate that Ag^+^ enables effective regulation of filament morphology and evolution, significantly improving film quality and surface uniformity under varying shallow defect conditions. Thermodynamic analysis reveals that Ag^+^ preferentially localizes at perovskite interfaces or surfaces, thereby avoiding undesirable aggregation within the lattice and maintaining structural stability. This interfacial positioning facilitates the reduction of Ag^+^ under applied bias, enabling controllable filament formation and rupture. Density functional theory and Green’s function calculations further confirm that Ag^+^ incorporation enhances electronic transparency near the Fermi level, consistent with experimental observations of ultralow set voltages and high ON/OFF ratios. This dual-pathway strategy—encompassing concurrent defect suppression and deep-level electronic modulation—provides a comprehensive framework for optimizing quasi-2D perovskite memristor design. Interstitially incorporated Ag^+^ not only facilitates microstructural refinement and controlled filament dynamics but also yields enhanced switching characteristics, with on/off ratios approaching ∼10^8^ and set voltages as low as ∼0.31 V, placing the device performance at the forefront of current quasi-2D perovskite-based memristor research. Overall, interstitial Ag^+^ not only optimizes the microstructure but also endows devices with greater tunability and stability, laying a solid foundation for future advances in high-performance neuromorphic and in-memory computing technologies.

## 4. Materials and Methods

### 4.1. Materials Preparation

All chemicals used in this study were purchased from commercial suppliers and used as received without further purification. Lead iodide (PbI_2_, 99.999%) and phenylethylammonium iodide (PEAI, 99.9%) were provided by Xi’an Yuri Solar Co., Ltd. (Xi’an, China). Silver iodide (AgI, 99%) was purchased from Sigma-Aldrich (St. Louis, MO, USA). Cesium iodide (CsI, 99.999%), N,N-dimethylformamide (DMF, anhydrous, 99.999%), and pre-patterned ITO/Glass substrates were purchased from Advanced Election Technology Co., Ltd. (Shenyang, Liaoning, China).

### 4.2. Device Fabrication

The Ag-free precursor solution was prepared by mixing PbI_2_, PEAI, and CsI in a molar ratio of 1:1:1 in anhydrous N,N-dimethylformamide (DMF, 99.999%). For Ag^+^-incorporated solutions, AgI was added to the above precursor solution with Pb:Ag molar ratios of 100:1, 100:5, and 10:1. The solutions were stirred at elevated temperature for more than 12 h (but less than 24 h). ITO/Glass substrates were sequentially cleaned by ultrasonic treatment in glass cleaner (240 min), acetone (20 min), and anhydrous ethanol (20 min), followed by oxygen plasma treatment to enhance wettability. The precursor solution was spin-coated onto ITO/Glass at 4000 rpm, followed by annealing at 150 °C for 180 s to obtain uniform perovskite films. The film fabrication process is illustrated in Figure S9.

To complete device fabrication, a 100 nm-thick Ag top electrode (30 μm × 30 μm) was thermally evaporated at a rate of 0.5 Å/s under a high vacuum ($1\times 10^{-6}$ Torr). Current–voltage (I–V) characteristics of devices with different Ag^+^ concentrations were measured; see Figure S10 for representative I–V curves.

### 4.3. Materials Characterization

The electrical properties of the memristor devices were measured using a custom-built test platform equipped with a Keithley 2450 source meter (Keithley Instruments, Cleveland, OH, USA) and a low-temperature probe station, both interfaced via a LabVIEW-based user interface. Surface and cross-sectional morphologies of the perovskite films were characterized using a field-emission SEM, ZEISS MERLIN COMPACT Carl Zeiss AG, Oberkochen, Germany. Film morphology and local conductivity were further investigated using AFM and C-AFM, Bruker Dimension Icon (Bruker, Billerica, MA, USA).

X-ray diffraction (XRD) patterns were recorded at room temperature using a Cu Kα radiation source (λ=1.5418 Å) on a BRUKER MILLER D8-Advance diffractometer. XRD data were collected in the 2θ range of 5–50° at a scan rate of 2° min^−1^. X-ray photoelectron spectroscopy (XPS, ULVAC-PHI GENESIS 900, ULVAC-PHI, Chigasaki, Japan) was performed to analyze the chemical states of Ag in the perovskite films.

Conductive AFM (C-AFM, Bruker) was used to probe changes in the local electrical properties of the perovskite films before and after Ag^+^ incorporation. Steady-state ultraviolet-visible (UV–vis) absorption spectra were collected on a PerkinElmer Lambda 1050+ UV–vis spectrophotometer (PerkinElmer, Waltham, MA, USA). XPS spectra were acquired with a PHI 5000 VersaProbe III spectrometer (ULVAC-PHI, Chigasaki, Japan) using Al Kα radiation (1486.6 eV).

Transient absorption (TA) spectra were measured using a femtosecond laser system (1050 nm, 1 kHz repetition rate, OPA, TA-ONE-1, TIME-TECH SPECTRA, Beijing, China) as the pump source, generating a probe wavelength of 420 nm.

### 4.4. DFT and Quantum Transport Calculations

All DFT calculations were carried out using the SIESTA package [45,46]. Norm-conserving pseudopotentials and a double-ζ polarized (DZP) basis set were employed, and the exchange-correlation interactions were treated within the generalized gradient approximation (GGA) using the Perdew–Burke–Ernzerhof (PBE) functional.

Electronic transport properties were investigated using the non-equilibrium Green’s function (NEGF) formalism, as implemented in the TranSIESTA module [47,48] of SIESTA. This approach enables the calculation of quantum conductance and electronic transmission spectra for two-probe device models under applied bias. All structures were fully relaxed until the forces were less than 0.02 eV/Å. The electronic temperature was set to 300 K. A *k*-point mesh of 2×2×50 was used along the transport direction, and a real-space grid cutoff of 300 Ry was applied. 

## Data Availability

Data will be made available upon request.

## Supplementary Materials

The following supporting information can be downloaded at https://www.mdpi.com/article/10.3390/nano15161267/s1.
