# Supplementary material for: Interstitial Ag+ Engineering Enables Superior Resistive Switching in Quasi-2D Halide Perovskites
Source: Nanomaterials (Basel). 2025 Aug 16;15(16):1267. doi: 10.3390/nano15161267 (PMC12389324; doi:10.3390/nano15161267)
Supplement: Supplementary file 1 [file nanomaterials-15-01267-s001.zip › nanomaterials-3777275-supplementary.pdf]

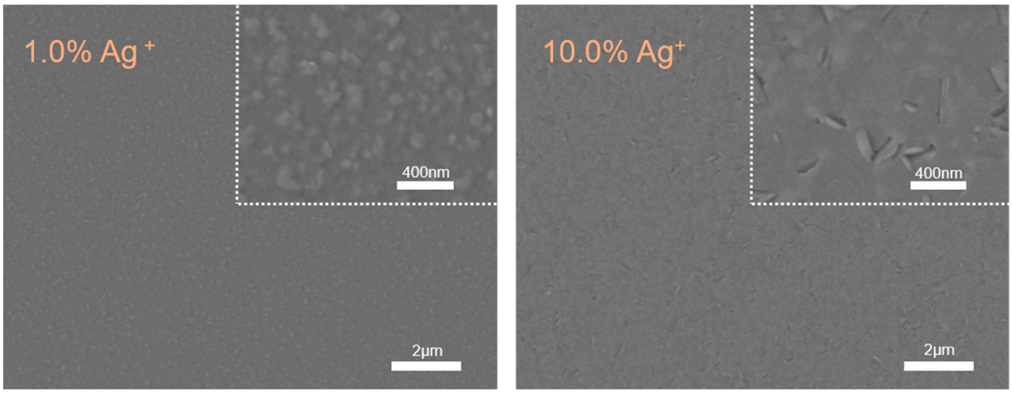

Figure S1. Scanning electron microscope (SEM) images of Q-2D perovskite films with various ratios of Ag^+.

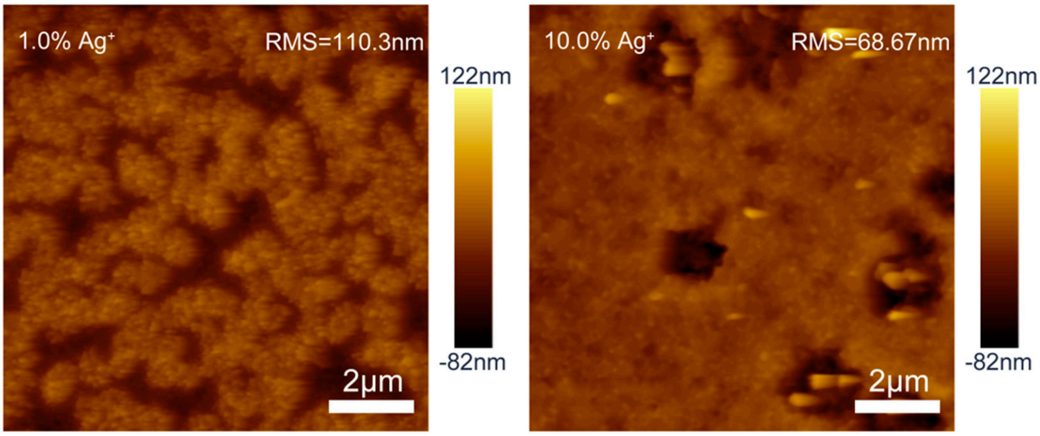

Figure S2. Atom force microscope (AFM) images of Q-2D perovskite films with various ratios of Ag^+.

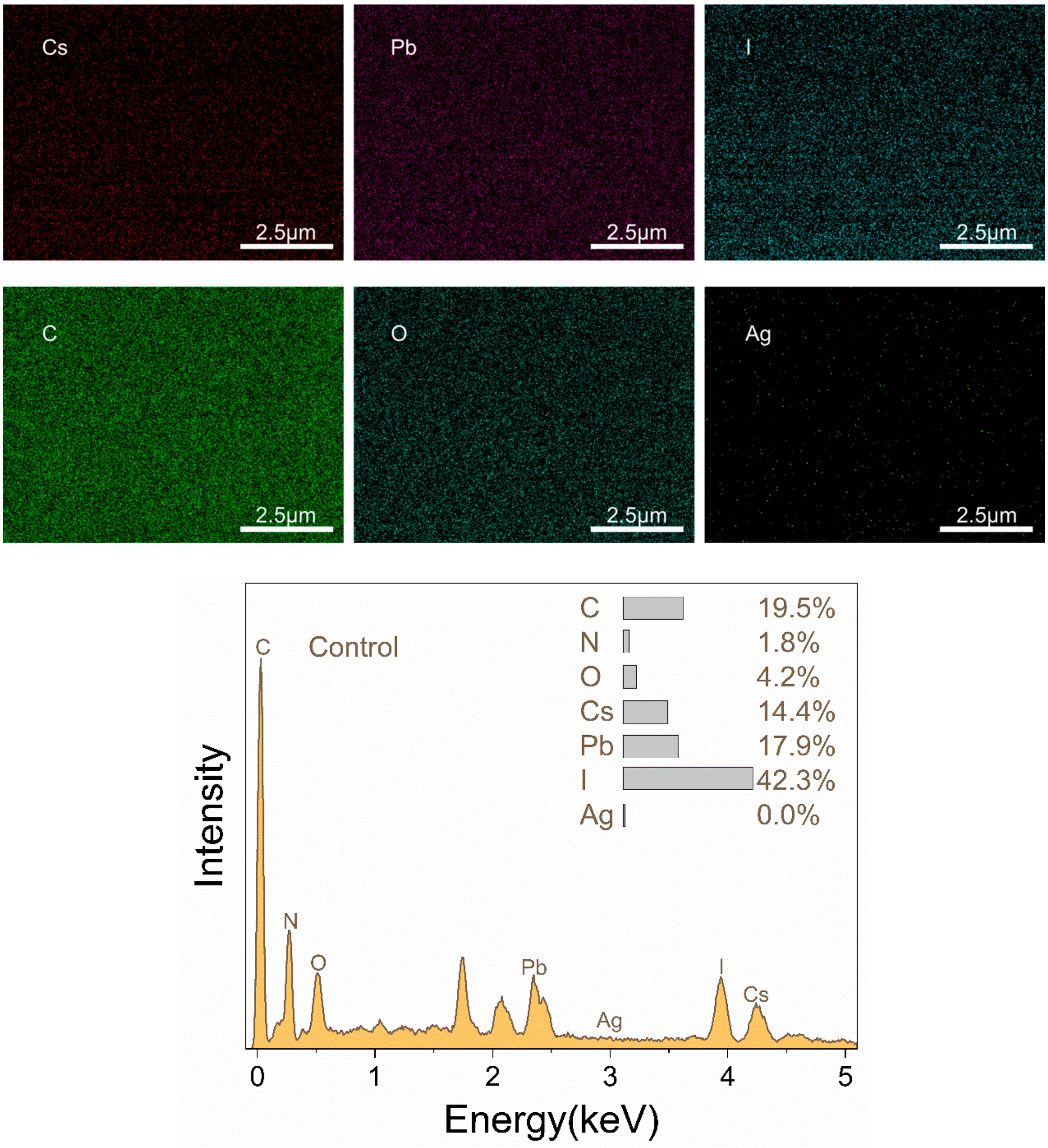

Figure S3. Energy Dispersive Spectroscopy (EDS) images and corresponding elemental distribution spectra of Q-2D perovskite films without Ag^{+}.

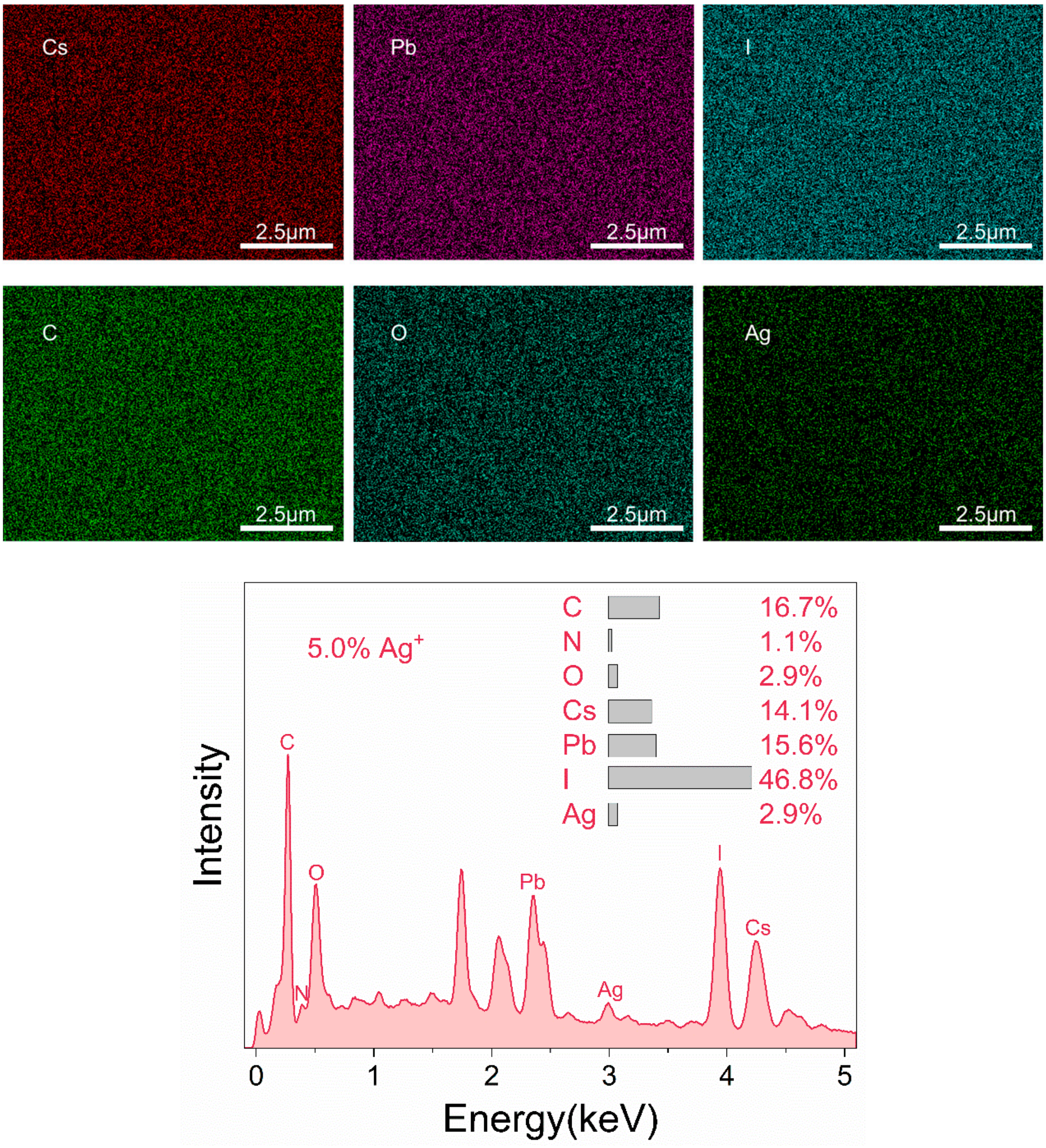

Figure S4. Energy Dispersive Spectroscopy (EDS) images and corresponding elemental distribution spectra of Q-2D perovskite films with Ag^+.

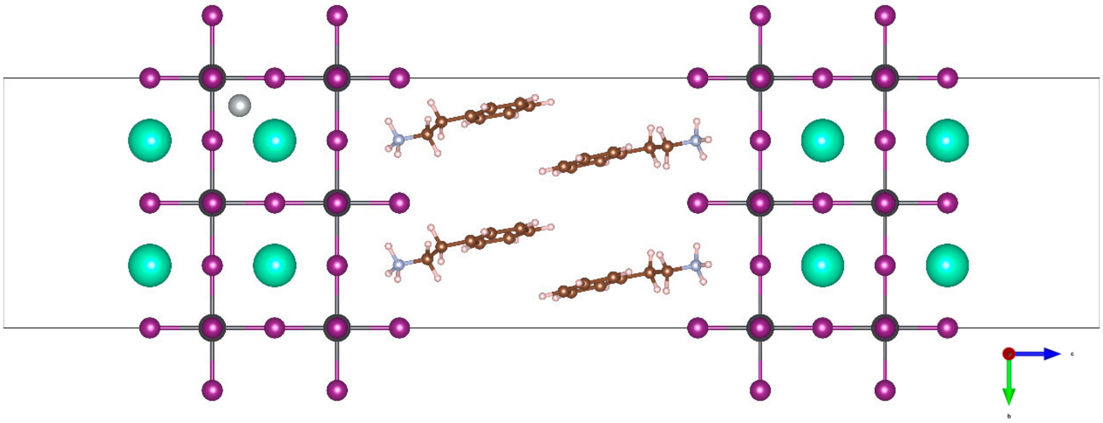

Figure S5. Structural schematic depicting Ag^{+} ion incorporation within the perovskite lattice.

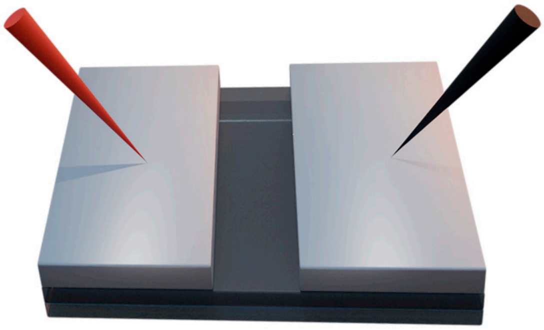

Figure S6. Schematic diagram of the device structure designed for conductive filament observation.

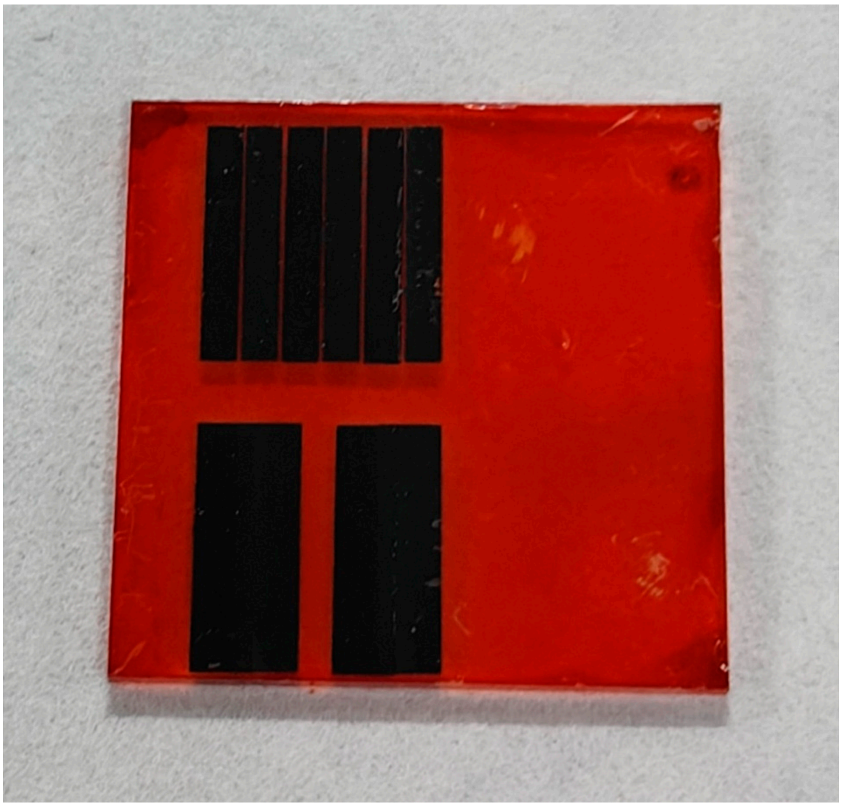

Figure S7 .Optical image of the device designed for for conductive filament observation.

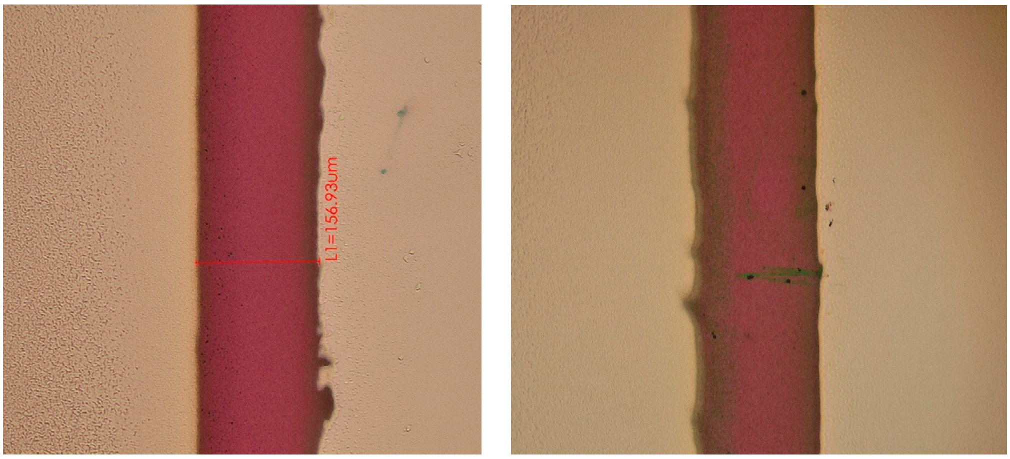

Figure S8. Optical microscopy image showing a suspected conductive filament region.

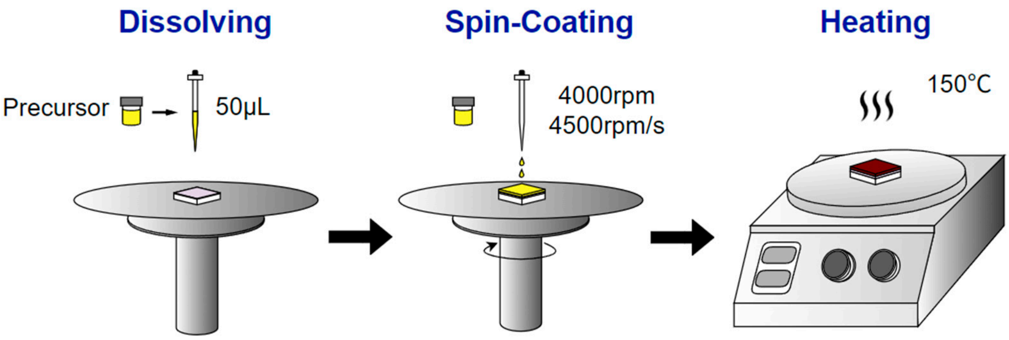

Figure S9. Diagram showing the preparation steps for perovskite films.

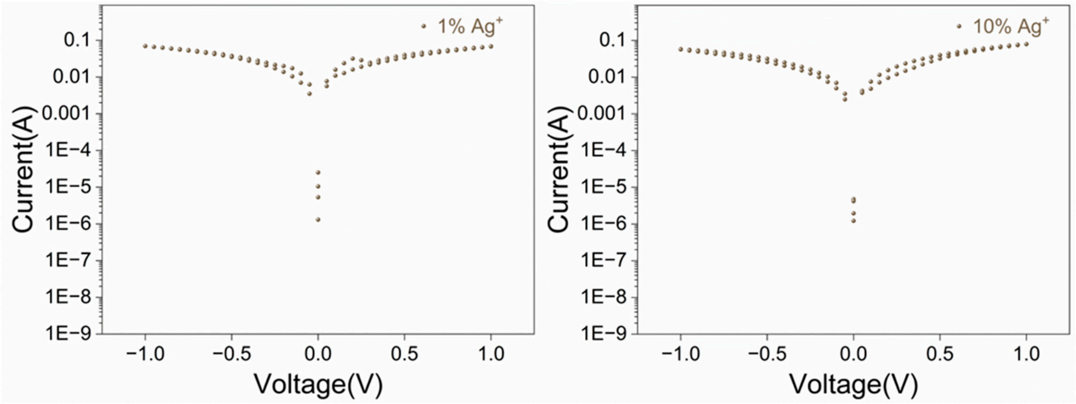

Figure S10. Current–voltage (I–V) characteristics of perovskite memristor devices with various ratios of Ag^{+}.

TableS1. Summary of TA dynamics spectra fitting parameters for control and 5.0%Ag^+ films.

**Sample**	**y_0**	**A_1**	**t_1(ps)**	**A_2**	**t_2(ps)**	**t_{ave}(ps)**
control	0.039809	0.05770	4.87445	0.40135	896.0437	894.7
5.0% Ag^+	0.07768	0.02626	1.16353	0.33724	1218.26947	1217.6

The decay dynamics of the sample (n=3,563nm) were analyzed by fitting the transient signal with a bi-exponential decay function, which can be expressed as:

$$\Delta A(t) = A_1 e^{-(t/t_1)} + A_2 e^{-(t/t_2)} + y_0 \qquad \text{(S1)}$$

where A_1 and A_2 are the amplitudes, t_1 and t_2 are the corresponding decay lifetimes, and y_0 is the baseline offset. This model captures both fast and slow decay processes associated with different excited-state species or mechanisms.The average lifetime t_{ave} is calculated using the following weighted formula, which accounts for both amplitude and decay constants of each component:

$$t_{ave} = (A_1 t_1^2 + A_2 t_2^2)/(A_1 t_1 + A_2 t_2) \qquad \text{(S2)}$$

This method provides a more accurate representation of the mean decay time, especially when multiple relaxation pathways are involved.

TableS2. Comparison of various halide perovskites -based devices.

Device structure	SET voltage [V_{set}]	ON/OFF ratio	Reference
Ag/Ca/PEA_2SnI_4/PI/Ag	0.2V	10^3	[11]
Ag/$Cs_3Bi_{2-x}LixI_{9-2x}$ (CBL_xI)/ITO (x = 0, 0.2, 0.4, 0.6)	0.1V	55	[17]
Ag/Al_2O_3/Cs–Cu–I/ITO	0.7V	10^3	[28]
Au/$Cs_2AgBiBr_6$/ITO	1.53V	10^3	[29]
Ag/NiO_x/$Cs_2AgBiBr_6$/SnO_2/ITO	0.3V	10^3	[30]
Ag/PMMA/$Cs_3Cu_2I_5$/ITO	0.34V	10^3	[31]
Pd/$MAPbI_3$/ITO	0.75V	10^3	[32]
Au/$(PEA)_2PbI_4$/FTO	4.2V	10^4	[33]
Ag/PMMA/$MAPbI_3$/FTO	|-0.1|V	10^7	[34]
Au/Ag/PMMA/OIHP:Ag/ITO/PEN	0.22V	10^4	[35]
Ag/Cs_2SnI_6/ITO	1.23V	2	[36]
Ag/$(H_2MPP)_2BiCuI_8$/ITO	0.4V	3.3	[37]
Al/$Cs_3Bi_2X_9$ (X = I, Br, and Cl)/ITO	0.1V	10^4	[38]
Ag/PMMA/$AgBiI_4$/ITO	0.16V	10^4	[39]
Ag/PMMA&$Cs_3Bi_2I_9$/ITO	0.6V	1200	[40]
Ag/ $(PEA)_2(MA)_{n-1}PbnI_{3n+1}$/ITO/PET	>0.6V	7×10^3	[41]
Ag/$BiCH_3NH_3PbI_3$/ITO	0.19V	10^2	[42]
Ag/AgI/$MAPbBr_3$/PEDOT:PSS/FTO	0.186V	10^3	[43]
Al/$Cs_3Cu_2Br_5$/ITO	0.45V	10^2	[44]
This Work	0.31V	10^8	/
